# Two Cases of Autoimmune Syndrome Induced by Adjuvants (ASIA): A Multifaceted Condition Calling for a Multidisciplinary Approach

**DOI:** 10.7759/cureus.30397

**Published:** 2022-10-17

**Authors:** Christine E Loftis, Aidee C Nunez, Mauricio De La Garza, Emilia Dulgheru

**Affiliations:** 1 Internal Medicine, University of Texas Rio Grande Valley School of Medicine, Edinburg, USA; 2 Plastic & Reconstructive Surgery Institute, Doctors Hospital at Renaissance (DHR Health), McAllen, USA; 3 Rheumatology Institute, Doctors Hospital at Renaissance (DHR Health), Edinburg, USA

**Keywords:** autoimmune hemolytic anemia, silicone breast implants, immune mediated necrotizing myopathy, breast implant illness, autoimmune syndrome induced by adjuvants

## Abstract

Silicone implants have been used for cosmetic enhancement and reconstructive purposes for over 60 years. Despite assiduous efforts to ensure safety, there is continuous evidence that they are not as biologically inert as previously postulated. We present two cases of autoimmune syndrome induced by adjuvants (ASIA) in Hispanic women. The first patient developed biopsy-proven immune-mediated necrotizing myopathy that was successfully treated with the combination of silicone explantation along with immunosuppressive therapy. Findings after implant removal demonstrated rupture and leakage of silicone from gluteal implants. The second patient developed autoimmune hemolytic anemia in the setting of a ruptured silicone breast implant. Similarly, the patient was treated with corticosteroids followed by breast implant removal with complete resolution of symptoms. The successful treatment of these patients was achieved by collaboration between rheumatology and plastic surgery, which emphasizes the need for a multidisciplinary approach in the diagnosis and management of patients with ASIA.

## Introduction

The use of silicone implants has been in practice since 1962 for cosmetic enhancement and reconstructive purposes [1]. The chemical structure and bioengineering of prosthetic breast implants continuously evolve to achieve the best safety and patient satisfaction and to avoid long-term complications [2]. Despite assiduous efforts, there is more evidence that silicone implants are not as biologically inert as previously postulated [3]. The emergence of breast implant illness (BII) or autoimmune syndrome induced by adjuvants (ASIA), composed of a vast array of clinical manifestations and immune abnormalities, led to a developing field of research that can only be further expanded by collaboration between plastic surgeons, rheumatologists, and immunologists. 

We present two cases of silicone implant-associated autoimmunity disorder in which the implants' removal in combination with immunosuppressive medications led to cessation of disease. 

## Case presentation

Case 1 

The patient was a 55-year-old Hispanic female with a history of breast and gluteal augmentation with silicone implants 24 years before presentation, followed by the exchange of breast silicone implants for saline implants 10 years afterward. She was referred to rheumatology by plastic surgery service for evaluation of autoimmune disorder secondary to silicone implants. Two years prior to the current presentation, and approximately 22 years after the original breast and gluteal implant augmentation, the patient developed paresthesia, Raynaud's phenomenon, dysphagia, and progressive proximal muscle weakness. The patient’s family history was notable for diabetes mellitus in her brother and mother, and there was no known history of any connective tissue or rheumatic diseases in the family. Physical examination was remarkable for objective proximal muscle weakness and active Raynaud's phenomenon. She had no sclerodermatous skin changes, rashes, or cuticular changes, and nailfold capillaroscopy was negative for vascular abnormalities. The patient was found to have elevated creatine phosphokinase (CPK), aldolase, and liver enzymes, and she had an initial positive antinuclear antibodies (ANA) of 1:640 with a nuclear pattern. The 11 myositis-specific antibodies panel was negative (Table 1).

**Table 1:** IMNM rheumatological laboratory studies at presentation ANA: antinuclear antibody; ds: double stranded; Ab: antibody; CRP: C-reactive protein; SS: Sjögren's syndrome; Scl: scleroderma; U1RNP: small nuclear ribonucleoprotein; CPK: creatine phosphokinase; AST: aspartate transaminase; ALT: alanine transaminase; ESR: erythrocyte sedimentation rate; SRP: signal recognition particle; HMGCR: 3-hydroxy-3-methylglutaryl-CoA reductase; MDA-5: melanoma differentiation-associated protein 5; NXP-2: nuclear matrix protein; IMNM: immune-mediated necrotizing myopathy

Parameter	Result	Reference Range
ANA	1:640	< 1:40
Anti-dS-DNA Ab	<1 IU/mL	< 10 IU/mL
Anti-Smith Ab	<1 IU/mL	< 10 IU/mL
Complement C3	81 mg/dL	87-200 mg/dL
Complement C4	18 mg/dL	19-52 mg/dL
CRP	4.69 mg/dL	< 1.0 mg/dL
Anti-histone Ab	< 1 IU/mL	< 25 IU/mL
Anti-Scl 70-Ab	< 7.0 U/mL	< 7.0 U/mL
Anti-SS-A Ab	< 7.0 U/mL	< 7.0 U/mL
Anti-SS-B Ab	< 7.0 U/mL	< 7.0 U/mL
Anti-centromere	< 7.0 U/mL	< 7.0 U/mL
Anti-U1RNP Ab	<1 U/mL	< 5.0 U/mL
Aldolase	59.9 unit/L	< 8.1 unit/L
CPK	5596 IU/L	30-223 IU/L
Myoglobin	1220 mcg/L	<66 mcg/L
AST	128 IU/L	13-39 IU/L
ALT	176 IU/L	7-52 IU/L
Anti-actin Ab	<20 IU/mL	<20 IU/mL
Anti-mitochondrial Ab	< 20 IU/mL	< 20 IU/mL
ESR	15 mm/h	0 to 22 mm/h
Anti- Pl-12 Ab	<11 IU/L	<11 IU/L Negative
Anti- Pl-7 Ab	<11 IU/L	<11 IU/L Negative
Anti- EJ Ab	<11 IU/L	<11 IU/L Negative
Anti-Jo-1 Ab	<11 IU/L	< 11 IU/L Negative
Anti- SRP Ab	<11 IU/L	<11 IU/L Negative
Anti- HMGCR Ab	<11 IU/L	<11 IU/L Negative
Anti-OJ Ab	<11 IU/L	<11 IU/L Negative
Anti-Mi-2 Alpha Ab	<11 IU/L	<11 IU/L Negative
Anti-Mi-2 Betta ab	<11 IU/L	<11 IU/L Negative
Anti-MDA-5 ab	<11 IU/L	<11 IU/L Negative
Anti-Tif-1 Gamma ab	<11 IU/L	<11 IU/L Negative
Anti-NXP-2 ab	<11 IU/L	<11 IU/L Negative

**Figure 1:**
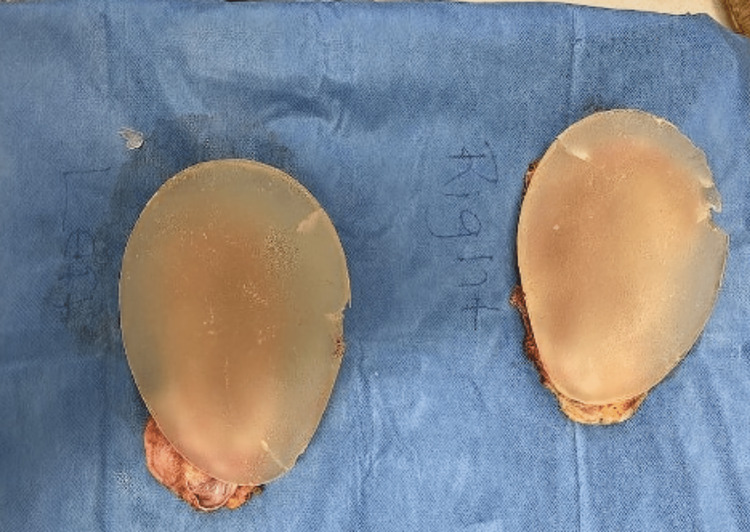
Gross pathology of bilateral silicone gluteal implants demonstrating fragmentation

**Figure 2:**
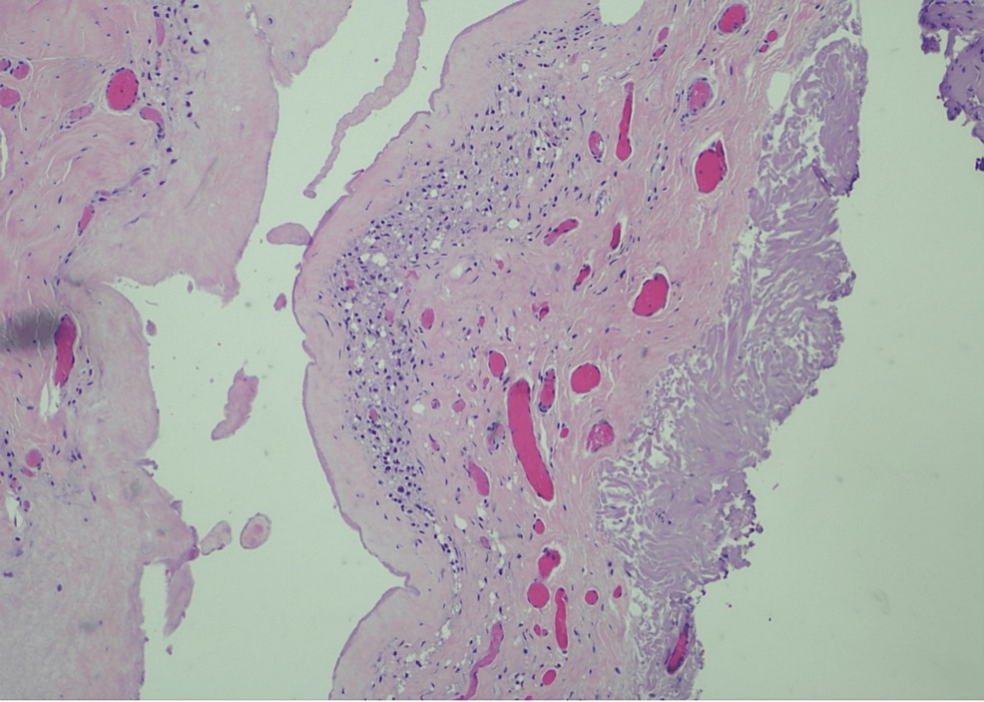
Histolopathology from gluteal implant demonstrating fibroadipose capsular tissue and skeletal muscle with scattered inflammatory cells

MRI of the thighs demonstrated signal abnormality in the posterior muscular compartment suggestive of myositis. Subsequently, the patient underwent muscle biopsy, which was remarkable for type 1 fiber predominance with atrophic type 2 muscle fibers and necrotic fibers with myophagocytosis, leading to a diagnosis of immune-mediated necrotizing myopathy (IMNM). The patient underwent treatment with prednisone 1 mg/kg and two months of intravenous immunoglobulins (IVIG) with only mild improvement of symptoms. Due to concern for ASIA, the patient underwent explantation of breast and buttock implants six months after the diagnosis of IMNM. While the saline breast implants were intact, bilateral silicone gluteal implants were ruptured (Figure 1). Both right and left gluteal tissue and capsule show skeletal muscle with scattered inflammatory cells (Figure 2). During dissection of the left buttock, a small area of murky, brownish-white fluid was found and was submitted to pathology for microbiology and cytology. The results of pathology revealed amorphous debris and mixed inflammatory cells and macrophages. Fluid cultures were negative. Around the time of explantation, the patient was re-evaluated by rheumatology and was started on mycophenolate mofetil (MMF) up to 1 gm twice daily and intramuscular steroids weekly with modest improvement of symptoms.

The patient continued to manifest muscle weakness and significant gastrointestinal symptoms, including heartburn, early satiety, abdominal distention, constipation, occasional nausea, and vomiting. She was further diagnosed with failure to thrive and pneumatosis intestinalis due to small intestinal bacterial overgrowth (SIBO). She was managed with prokinetics, including metoclopramide, erythromycin, and rifaximin. She was started on intravenous immunoglobulin (IVIG) 1 gm/kg X 2 days monthly and IV corticosteroids, 1 mg/kg in divided doses. Due to severe weight loss, she underwent percutaneous gastrostomy (PEG) placement and was started on TPN. Nine months after explantation, she continues with MMF and IVIG monthly and has tapered off corticosteroids. Muscle strength has improved progressively, she no longer has Raynaud's phenomenon, and she is demonstrating continuous improvement of GI symptoms, tolerating a soft diet and continuing total parenteral nutrition (TPN) intermittently. CPK and aldolase returned to normal and repeat ANA and 11 myositis panels remained negative on multiple occasions. 

Case 2 

**Figure 3:**
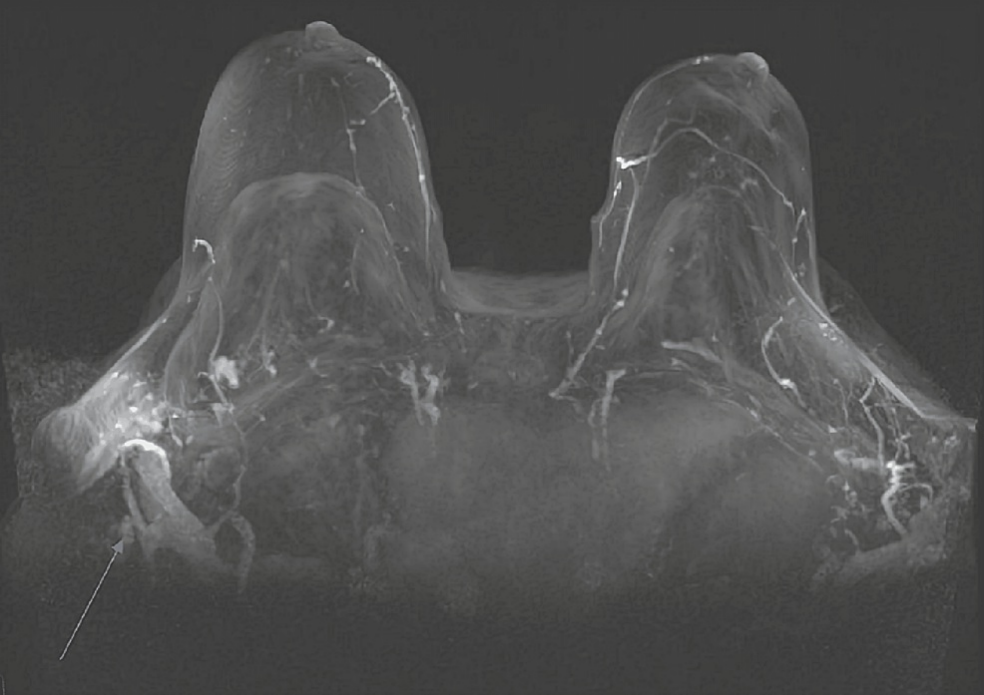
MRI of the breast was remarkable for right breast implant rupture with silicone lymphadenitis (White arrow)

A 73-year-old Hispanic female with a history of silicone breast augmentation for cosmetic purposes 18 years before the presentation was referred to rheumatology with a diagnosis of autoimmune hemolytic anemia (AIHA). The patient did not have a family history of autoimmune conditions but did have a strong family history of cancer including a father with lung cancer, a sister with breast cancer, and a brother with gastric cancer. The patient first noticed swelling in the supraclavicular area and developed "big lumps" on the top of the clavicle that started eight months before the presentation. She mentioned the lumps were intermittently "turning very red." The mammogram was remarkable for several scattered echogenic masses casting posterior snowstorm artifacts identified along the right infraclavicular soft tissues, axilla, and internal mammary chain consistent with extracapsular silicone with rupture suspected. MRI of the breast was remarkable for right breast implant rupture with silicone lymphadenitis (Figure 3). The patient underwent fine needle aspiration of supraclavicular mass for cytology. Specimen comprised a few mixed acute and chronic inflammatory cells, macrophages, and amorphous granular debris. No malignant cells were identified. The fluid was negative for silicone particles. The patient was diagnosed with AIHA given a direct antiglobulin test (DAT) that was IgG positive, and C3D positive, and underwent extensive hematologic workup for etiological diagnosis of AIHA (Table 2). The patient was managed for AIHA with prednisone 1 mg/kg and was further referred to rheumatology. Clinically, she did not manifest symptoms worrisome for connective tissue disease. 

**Table 2:** AIHA case hematologic labs at presentation MCV: mean corpuscular volume; LDH: lactate dehydrogenase; DAT: direct antiglobulin test; SPEP: serum protein electrophoresis; AIHA: autoimmune hemolytic anemia

Parameter	Result	Reference Range
Hemoglobin	8.4 gm/dL	10.8 gm/dL
Hematocrit	25.9 %	32.2 %
MCV	109.3 fl	84 fl
Percent reticulocyte	18.08 %	0.7- 2.8%
Absolute reticulocyte	0.47 m/uL	0.00-0.15 m/uL
Total bilirubin	3.2 mg/dL	0.2 -1.2 mg/dL
Indirect bili	2.4 mg/dL	0.2 -1.2 mg/dL
Haptoglobin	< 8 mg/dL	43-212 mg/dL
LDH	526 IU/L	140-271 IU/L
B 12 Level	250 nmol/L	
DAT	IgG positive, CD3 positive	
SPEP	No monoclonal protein present	
Peripheral blood smear	RBC with numerous microspherocytes and polychromatophilic cells	

**Figure 4:**
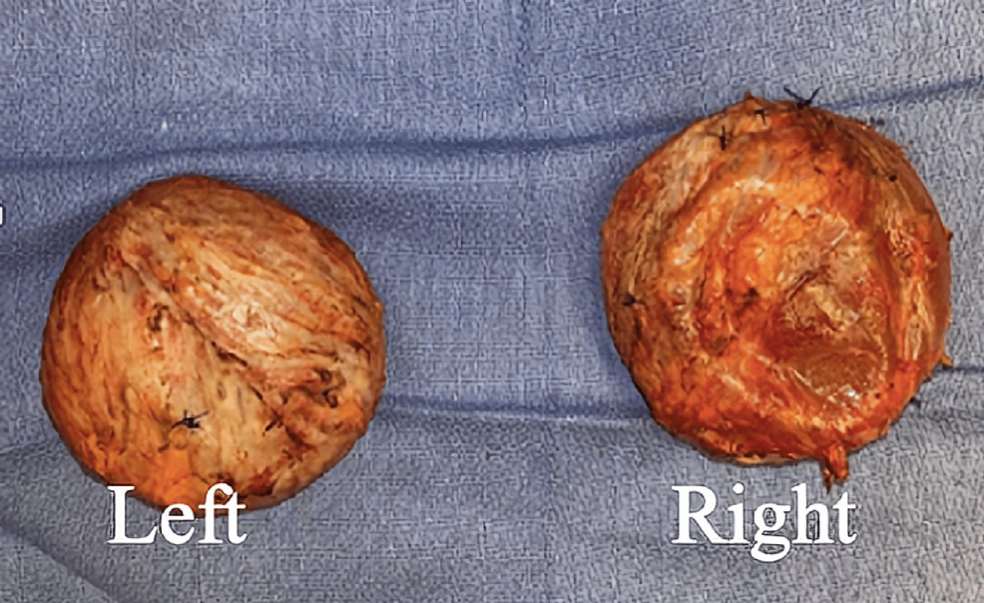
Gross pathology demonstrating bilateral breast implant with capsule

**Figure 5:**
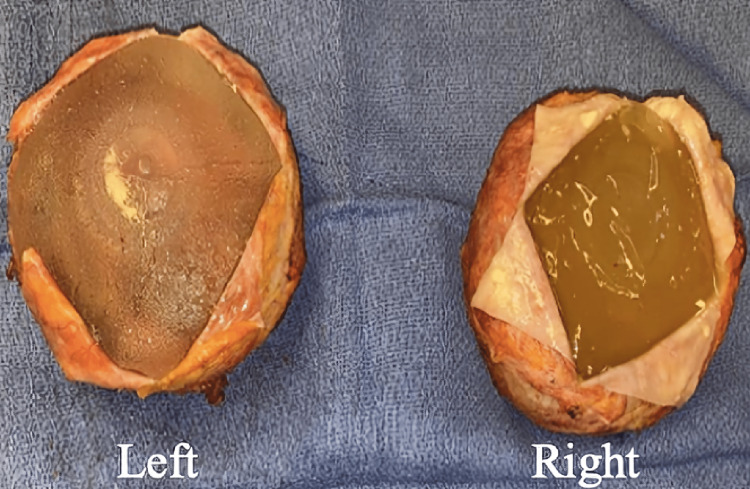
Gross pathology demonstrating right collapsed open capsule

**Figure 6:**
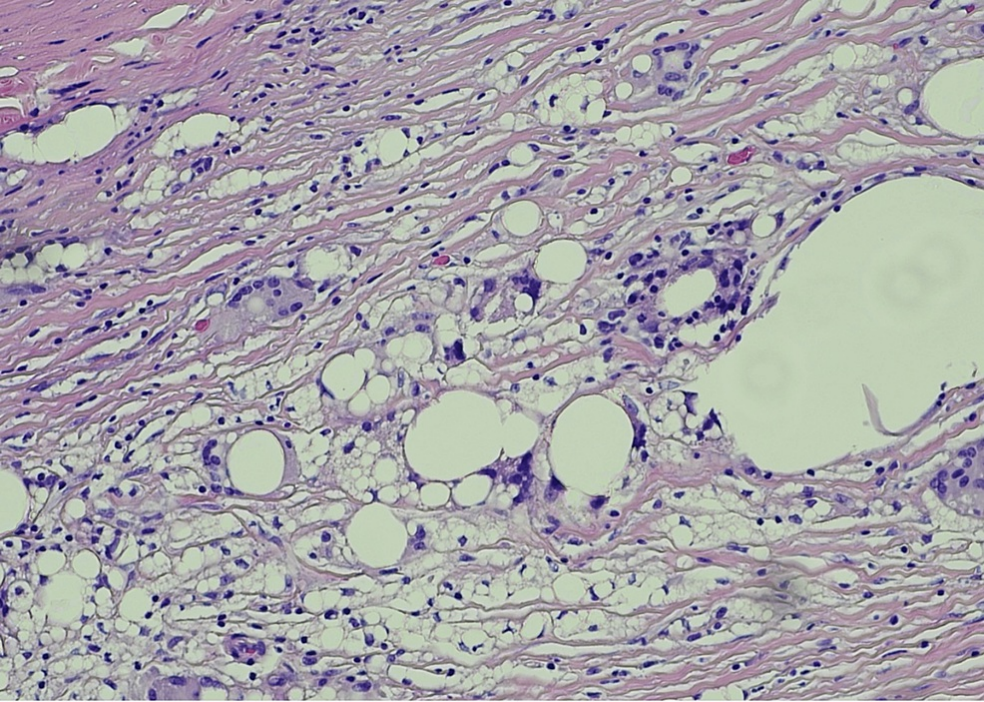
Right breast implant and capsule tissue (magnification X 20) showing patchy inflammation, focal fat necrosis, and focal foreign body cell reaction around silicone particles

ANA was positive with a titer of 1:1280 by IF. Another autoimmune workup was negative (Table 3). A repeat ANA by immunofluorescence was positive with a titer of 1:320 by IF and anti-histone antibodies were negative. Given the patient's clinical presentation along with the timing of the silicone breast rupture, a diagnosis of ASIA was made, and the removal of silicone implants was recommended. The patient underwent the procedure, and the biopsy was consistent with a right collapsed silicone breast implant while microscopy revealed benign fibroadipose (capsular) tissue, patchy inflammation, focal fat necrosis, and focal foreign body giant cell reaction (Figures 4, 5, 6). There was no evidence of malignancy and the left breast had an intact-unruptured silicone implant (Figure 5). One week after explantation, corticosteroids were discontinued, the patient did not require additional treatment, and her hemoglobin and hematocrit improved postoperatively to 12.5 gm/dL and 35.1 % and remained stable 17 months post-explantation. 

**Table 3:** AIHA case rheumatological labs at presentation ANA: antinuclear antibody; ds: double stranded; Ab: antibody; CRP: C-reactive protein; SS: Sjögren's syndrome; Scl: scleroderma; U1RNP: small nuclear ribonucleoprotein; ANCA: antineutrophil cytoplasmic antibodies; MPO: Myeloperoxidase; RNP: ribonucleoprotein; AIHA: autoimmune hemolytic anemia

Parameter	Result	Reference Range
ANA	1:1280	< 1:40
Anti-dS-DNA Ab	0.6 IU/mL	< 10 IU/mL
Anti-histone Ab	< 1.0 unit	
ANCA screen	Negative	Negative
Anti-MPO Ab	<1.0 AI	< 1.0 AI
Anti-PRO-3 ab	<1.0 AI	< 1.0 AI
Complement C3	102 mg/dL	87-200 mg/dL
Complement C4	18 mg/dL	19-52 mg/dL
CRP	0.6 mg/dL	< 1.0 mg/dL
Anti-TPO ab	<4.0 IU/mL	< 25 IU/mL
Anti-Jo-1 ab	<0.3 U/mL	< 7.0 U/mL
Anti-Scl70-ab	0.8 U/mL	< 7.0 U/mL
Anti-SS-A ab	0.4 U/mL	< 7.0 U/mL
Anti-SS-B ab	<0.3 U/mL	< 7.0 U/mL
Anti-Centromere	4.8 U/mL	< 7.0 U/mL
Anti-U1RNP ab	1.6 U/mL	< 5.0 U/mL
Anti-RNP-70 ab	<0.3 U/mL	< 7.0 U/mL
Anti-Mi-2 ab	<20.0 unit	<20.0 unit

## Discussion

Silicone polymers were introduced in plastic and reconstructive surgery in 1962, but the first report of their use was as a waterproof wound dressing in 1947 [4]. Polymers of silicone have been designed as a material immunologically inert and provide a consistency similar to human tissues [5]. However, more recent data demonstrate that silicone implants are not as innocuous as initially theorized [3]. 

While local host reactions secondary to silicone breast implants, including capsule formation, capsular contracture, implant rupture, and local pain, are well described in plastic and reconstructive surgery literature, the characterization of systemic autoimmune reactions associated with silicone remains under scientific grapple. From the rheumatology perspective, some patients with known autoimmune conditions have been retrospectively identified to have undergone silicone implants [6]. The implant could have been a potential trigger of autoimmunity in genetically predisposed individuals. Of note, patients frequently omit to include cosmetic procedures in their past medical history as these are considered elective and not for medical purposes [4]. Although plastic surgeons are well-versed in diagnosing and managing local reactions associated with silicone implants, they might not be as familiar with the systemic effects of silicone. 

While plastic surgery literature coins the term breast implant illness (BII), the immunology and medical literature often uses the term ASIA and classifies the autoimmune reaction secondary to silicone under the ASIA umbrella. Silicone implant incompatibility syndrome is another term used in the literature to describe this condition [7]. 

Given the myriad clinical presentations, discordant nomenclature, and lack of definitive classification criteria for the syndrome, diagnosis and management may present a dilemma for both specialties. This article described two patients with a history of silicone implant augmentation that developed an autoimmune condition, successfully co-managed with collaborative efforts from rheumatology, pathology, radiology, and plastic surgery. 

The first patient had initial silicone breast and gluteal implants 24 years before the presentation and exchange for saline breast implants 10 years before the presentation. She was diagnosed with immune-mediated necrotizing myopathy. The disease remained active even after the explantation of breast and gluteal implants. She required an intense and prolonged immunosuppressive regimen lasting for approximately six months in order to achieve remission. The second patient had an 18-year history of silicone implants and presented with AIHA, which required corticosteroid treatment and removal of ruptured silicone implants. AIHA remained in remission without any immunosuppression 17 months post explantation and did not require any treatment post explantation. 

Patients with ASIA can present with multiple non-specific complaints, including fatigue, sleep disturbances, cognitive impairment, arthralgias, myalgias, dry eyes and mouth, rashes, and fever that fail to reach the criteria for a defined rheumatological disease. Other patients have clear clinical and serologic manifestations that can be classified as Sjogren's syndrome, systemic sclerosis, rheumatoid arthritis, or sarcoidosis [8,9]. As per the literature, only a few reported cases of AIHA and respectively inflammatory myopathy have been associated with silicone implants [10,11]. 

The patient with IMNM was initially managed with immunosuppressive medications with minimal improvement. Due to the concern of ASIA, the decision to remove all implants was made. The gluteal implants were fragmented (Figure 1), and the breast saline implants were intact. Similarly, in the second case, the AIHA was diagnosed concomitantly with silicone implant rupture that manifested with intense local reaction. (Figure 3, 6) 

As the implant shell elastomer ages, it eventually fails, leading to "silicone bleeding," described as the migration of low molecular silicone core particles through the high molecular silicone shell. Furthermore, the shell rupture facilitates this process [8,12,13]. The presence of polydimethylsiloxane has been identified at sites distant from the implant [13]. In our patient with AIHA, the imaging studies identified silicone in the right infraclavicular soft tissues, axilla, and internal mammary chain lymph nodes described as "snowstorm artifacts". Even though it remained unproven, the supraclavicular fluid aspirate could have potentially contained silicone particles. Of note, the supraclavicular swelling resolved post treatment. It is important to mention that silicone may be hard to identify by conventional light microscopy techniques in histopathologic specimens and special stains may be needed and should be specifically requested [14] 

It has been postulated that as the implant elastomer ages, silicone can trigger macrophage activation, leading to further histiocytic reaction with siliconomas including at distant sites. Interestingly, our first patient presented with immune-mediated necrotizing myopathy, a disease characterized by a scant inflammatory infiltrate of a predominant monocytic lineage [15]. It is perhaps important to note the proximity of the gluteal implants to thigh muscle extensors as well as the inflammatory infiltrate that was aspirated from the periprosthetic area. Furthermore, multiple case reports of sarcoidosis induced by silicone implants have been identified in the literature [16]. 

To our knowledge, this is the first case of AIHA associated with silicone implant rupture. In animal studies, the injection of silicone gel in New Zealand Black (NZB) mice has led to the induction of proteinuria and AIHA [17]. Furthermore, silicone-associated illness has been reported in patients with a specific genetic predisposition, with vitamin-D deficiency, smokers, and patients with immunoglobulin deficiency [8,18]. 

## Conclusions

Between questioning its existence to inconsistent naming and heterogenous presentation, silicone-induced disease calls for a multidisciplinary approach, including plastic surgeons, immunologists, radiologists, pathologists, and rheumatologists. This collaboration may further help close the gap in defining this heterogenous group of manifestations, clinical presentation, pathogenesis, and nomenclature as well as definitely prove causation. Proper identification of these patient groups through a multidisciplinary approach before implantation may help with risk stratification. Management is also at the frontier of two specialties. As illustrated in our first case, the patient continued to have symptoms despite silicone removal, which correlates with findings in the literature that explantation alone does not lead to the reversal of the disease process and that combined immunosuppressive therapy and prosthesis removal are vital in treatment. Timing of surgical explantation as well as the use of immunosuppressive medications in view of their potential deleterious postsurgical outcomes should be also a topic of collaboration between the specialties. These findings further support the need for multidisciplinary management of these complex patients. 

Our article aims to continue increasing awareness and open the collaboration between plastic surgeons and rheumatologists to treat potential cases of breast implant illness in a multidisciplinary manner. Risk stratification preimplantation may further identify patients that are at risk of developing ASIA syndrome. 
